# Health-related quality of life in adults with epidermolysis bullosa: a cross-sectional study in seven European countries using EQ-5D-5L

**DOI:** 10.1186/s13023-026-04299-1

**Published:** 2026-03-19

**Authors:** Renata Linertová, Márta Péntek, Benjamín Rodríguez-Díaz, Christine Bodemer, Vinzenz Hübl, May El Hachem, Gudrun Salamon, Verónica Alonso-Ferreira, Georgi Stefanov, Ritu Jain, Yolanda Ramallo-Fariña, Lidia García-Pérez, BUR-EB Study Group

**Affiliations:** 1 Fundación Canaria Instituto de Investigación Sanitaria de Canarias (FIISC), Santa Cruz de Tenerife, Spain; 2 Network for Research on Chronicity Primary Care and Health Promotion (RICAPPS), Tenerife, Spain; 3 Health Economics Research Center (HECON), University Research and Innovation Center, Obuda University, Budapest, Hungary; 4 Doctoral School of Innovation Management, Obuda University, Budapest, Hungary; 5 Pediatric Dermatology Department and National Expert Centre for Genodermatoses (MAGEC-Necker), Necker Enfants Malades Hospital, APHP, University Paris Cité, Paris, France; 6 Department of Dermatology, Medical Center, Faculty of Medicine, University of Freiburg, Freiburg, Germany; 7 Dermatology Unit and Genodermatosis Research Unit, Translational Paediatrics and Clinical Genetics Research Area, Bambino Gesù Children’s Hospital, IRCCS, Rome, Italy; 8 HEALTH Lab | Competence Center for Medical and Health Psychology, Sigmund Freud University, Vienna, Austria; 9 Institute of Rare Diseases Research (IIER), Instituto de Salud Carlos III (ISCIII), Madrid, Spain; 10 Centre for Biomedical Network Research on Rare Diseases (CIBERER), Instituto de Salud Carlos III, Madrid, Spain; 11 Bulgarian Association of Promotion of Education and Science (BAPES), Institute for Rare Diseases, Plovdiv, Bulgaria; 12 Department of Social Medicine and Health Management, Faculty of Public Health Medical University, Pleven, Bulgaria; 13 DEBRA International, Vienna, Austria; 14 Lee Kong Chian School of Medicine, Nanyang Technological University, Singapore, Singapore

**Keywords:** Epidermolysis bullosa, Health-related quality of life, Patient reported outcomes, Health utilities, EQ-5D-5L

## Abstract

**Background:**

Epidermolysis bullosa (EB) is a rare genetic disorder that causes extreme skin fragility, chronic pain, and functional impairment, with major psychosocial and economic consequences. Health-related quality of life (HRQoL) data is critical to capture the full burden of EB. Health utilities derived from preference-based generic instruments such as the EQ-5D-5L provide standardized health status utility values that enable cross-disease comparisons and provide input data for cost-utility analyses to inform resource allocation. There is a notable lack of multinational, up-to-date utility data for EB. This cross-sectional study aimed to assess HRQoL in adults with EB across seven European countries (Austria, Bulgaria, Germany, Hungary, Italy, France, and Spain) using the EQ-5D-5L.

**Results:**

A total of 328 adults with EB participated in the survey, 61% were female, 37% were between 18 and 30 years old and 46% had dystrophic EB. Based on self-reported symptoms, 58% were classified as severe EB. Pain/discomfort was the most affected EQ-5D-5L dimension (92% reporting problems; 27% severe or extreme). The mean EQ-5D value (health utility) was 0.63 (SD 0.32), ranging from 0.57 in Spain to 0.71 in Bulgaria. Patients with severe EB reported significantly lower utilities than non-severe cases (0.52 vs. 0.78, *p* < 0.001). Mean EQ VAS score was 60 (SD 23.2). Compared to general population norms, EB patients in all countries had markedly lower HRQoL (*p* < 0.005), with large effect sizes for the EQ-5D value (Cohen’s *d* ≥ 0.8). Symptomatic burden and functional deterioration were the primary drivers of HRQoL impairments.

**Conclusions:**

This multinational study provides the most extensive and current health utility data for adults with EB in Europe. Findings reveal the profound HRQoL impairment in EB, particularly in severe cases. These standardized utility values fill a major evidence gap, supporting their use in health economic evaluations, cross-disease comparisons, and policy development.

**Supplementary Information:**

The online version contains supplementary material available at 10.1186/s13023-026-04299-1.

## Introduction

Epidermolysis bullosa (EB) is a group of rare genetic disorders characterized by extreme skin fragility, leading to chronic pain, recurrent wounds, and functional limitations [1]. The primary manifestation is the formation of blisters and erosions in response to mechanical trauma. Four main clinical types are recognised—EB simplex, junctional EB, dystrophic EB, and Kindler EB—each defined by the level of skin cleavage, but with broad variability in severity, prognosis, and associated complications [2]. The onset is typically at birth or in early infancy, and there is currently no curative treatment. Clinical care focuses on symptom relief and prevention of complications such as chronic wounds, malnutrition, joint contractures, squamous cell carcinoma, and functional disabilities [1]. The burden of EB extends far beyond the physical symptoms, encompassing persistent pain, significant functional limitations, and considerable psychosocial and economic impacts on both patients and caregivers [3–5].

Health-related quality of life (HRQoL) has emerged as a crucial endpoint in rare disease research. There is growing recognition that such data from patients provides important information that complements the clinical endpoints traditionally used in medical care, and can pick up problems and issues missed by them [6]. Disease-specific HRQoL instruments can offer deeper insights into particular conditions and support medical decision making in everyday practice. Preference-based generic HRQoL tools, such as the EQ-5D, are particularly valuable as they provide standardized health status utility values (i.e., they convert patient-reported health states into a single index score, called ‘value‘ or ‘utility’, reflecting societal preference for the given health state) that permit for comparisons among conditions, support cost-utility analyses and inform healthcare resource allocation, which is particularly relevant in rare diseases with high treatment burden such as EB [7].

The EQ-5D-5L (EQ-5D version with five-level response scale) has been extensively used in economic evaluations and demonstrated validity and responsiveness across a broad spectrum of conditions and age groups, including some rare disorders, supporting its feasibility for capturing general health status [7, 8]. Nevertheless, its use in EB populations remains extremely limited, with only a small number of national studies [9–11], and the most recent international data dating back over a decade [12]. For national economic evaluations, using EQ-5D utility values derived from other countries may introduce bias [13], as they reflect the health preferences of a different general population, which may not align with those of the population in which the economic evaluation is conducted. Furthermore, previous research has often used the earlier 3-level version (EQ-5D-3L), reported incomplete results, or lacked consistent utility value derivation, limiting comparability across studies and over time.

This scarcity of standardised, up-to-date, multinational utility data represents a significant gap in the evidence base, restricting the integration of HRQoL into broader health economic modelling and policy discussions. The present cross-sectional study aimed to assess HRQoL in a large cohort of adult EB patients from seven European countries using the EQ-5D-5L.

## Methods

### Study design and participants

This was a cross-sectional, observational study within the BUR-EB Project, an initiative promoted by the European Joint Programme on Rare Diseases (EJPRD), with the aim to quantify the socio-economic impact of EB on patients and families in Europe. It was carried out in seven European countries (Austria, Bulgaria, Germany, Hungary, Italy, France, and Spain) in collaboration with patient advocacy organizations (PAO) - DEBRA International and regional DEBRA associations, national EB registries and reference centres. In this paper we report analyses of EQ-5D-5L data gathered among adult patients as part of the dataset. Approvals from ethics committees and informed consent from participants were obtained in all countries.

A questionnaire was developed in English and translated into the relevant languages by the research teams in each participating country and subsequently reviewed by local EB experts, including representatives from patient organizations and healthcare professionals with expertise in EB. In addition to linguistic translation, the questionnaire was culturally adapted to account for country-specific aspects. The survey was distributed in on-line and printed versions by DEBRA associations, medical reference centres, dermatology clinics or through registries’ databases. The survey was promoted via direct emails and on social media of involved entities, and regular reminders were sent out. Non-institutionalized patients, residents in one of the participating countries, diagnosed with any type of EB were invited to participate and they could complete the questionnaire anonymously by themselves or with help of a caregiver or family member. The current paper report only adult patients’ results. No exact demographic data were asked in order to maintain the anonymity of the respondents, e.g. no exact age was asked, but only age range. The recruitment process was coordinated by DEBRA International over a period between June to September 2024.

### Health-related quality of life (HRQoL)

HRQoL was assessed by the EQ-5D-5L questionnaire, using official language versions provided by the EuroQol Group. The first part of the EQ-5D-5L (so called descriptive system), describes dimensions of mobility, usual activities, self-care, pain & discomfort and anxiety & depression; with five possible response levels expressed as no, mild, moderate, severe and extreme problems or unable to [6]. Participants are asked to indicate the response that best describes their current status. The combination of the responses under each dimension describes a person’s EQ-5D self-reported health state, called EQ-5D profile. A scoring system (country- or region-specific value sets), based on preferences of the general public, converts profile data to a single number - also called health utility or EQ-5D value. Utilities lie on a scale between 1, full health, and 0, death. Values less than 0 are possible for health states considered to be worse than dead. Country-specific value sets were used for Germany [14], France [15], Hungary [16], Italy [17] and Spain [18]; for Austria and Bulgaria there is no specific value set, therefore supra-national value sets (Central Western and Eastern European) were used [19]. Central Western value set was chosen to be used for overall sample, as most of the participants (66%) resided in this region.

The second part of the questionnaire is a visual analogue scale (VAS), called EQ VAS, which captures the respondent’s overall assessment of their current health on a scale from 0 (the worst imaginable health state) to 100 (the best imaginable health state).

A proxy version of EQ-5D-5L was used where EB patients were not capable to report their health status themselves, hence results reflect their close caregivers’ perspective.

### Analysis

As EB is a rare condition, the sample size was defined by the practical limits of patient recruitment instead of a formal power analysis. This strategy aligns with typical rare disease research, where small cohorts are unavoidable due to the limited number of patients. Only those responses that answered all questions relevant for this HRQoL study were analysed. Incomplete questionnaires were excluded.

To assess disease severity for the purposes of this analysis, we used 15 symptoms of EB included in the questionnaire (Tables S1A and S1B in the Supplementary material). Four EB experts identified eight of these symptoms as present in severe cases (pain, chronic wounds, malnutrition, joint deformities, functional disabilities, squamous cell carcinoma, gastrostomy, narrowed oesophagus). We then defined “severe” EB cases as those in which pain and at least one of the other seven symptoms were present; the rest of cases were defined as “non-severe”. Results are reported by country, EB type (dystrophic, EB simplex, junctional, kindler), severity (non-severe/severe), and for the entire study population.

Categorical variables were summarized using frequencies and percentages, while continuous variables were expressed as means and standard deviations. Group comparisons for categorical variables were performed using Fisher’s exact test or the Chi-square test, as appropriate. For comparisons of continuous variables across groups, the Kruskal–Wallis test was applied. Comparisons between the EQ-5D values and EQ VAS scores from study sample and population norms in each country [20–23] were conducted using *t*-tests. Effect sizes were calculated using Cohen’s *d* and differences of 0.2, 0.5, and 0.8 were considered small, moderate, and large effect sizes, respectively [24]. A p-value < 0.05 was considered statistically significant.

Univariate and multivariate multiple linear regression models were constructed to identify factors independently associated with the EQ-5D health scores. Age, sex, education and symptoms variables were considered as covariates under the significance criterion of *p* < 0.15.

All analyses were conducted using R v4.5.1.

## Results

### Sample characteristics

A total of 328 adult people with EB were included in the analysis (61% female; 37% were between 18 and 30 years old and 34% were between 31 and 50 years old). Initially, all received responses (*N* = 604) were thoroughly checked in order to include all valid ones (*N* = 346) but to exclude any duplicates (i.e., those who have completed the online survey twice; *N* = 18).

Most frequent EB types were dystrophic (45.7%) and simplex (29.3%), and about a half of the sample had generalized form of EB, although some patients did not know or report their EB type or subtype. Skin blisters was the most frequent symptom (95.4%), followed by pain (76.5%) and skin crusts (65.2%) (see Tables S1A and S1B in the Supplementary material for details on other symptoms). Based on reported symptoms, 58.2% of the patients were considered severe EB cases for this analysis and these patients reported statistically significantly higher number of all symptoms, except for skin blisters (Table S1B in the Supplementary material). No statistically significant differences were observed between countries regarding socio-demographic variables, except for education level (Table 1).

**Table 1:** Sample characteristics by country

Variable	Overall *n* = 328	Austria *n* = 33	Bulgaria *n* = 18	France *n* = 125	Germany *n* = 58	Hungary *n* = 18	Italy *n* = 45	Spain *n* = 31	*p*-value (between countries)
**Sex**,** n (%)**									0.568^b^
Female	200 (60.98)	18 (54.55)	12 (66.67)	74 (59.20)	38 (65.52)	8 (44.44)	31 (68.89)	19 (61.29)	-
Male	127 (38.72)	15 (45.45)	6 (33.33)	51 (40.80)	20 (34.48)	10 (55.56)	14 (31.11)	11 (35.48)	-
Other	1 (0.30)	0 (0.00)	0 (0.00)	0 (0.00)	0 (0.00)	0 (0.00)	0 (0.00)	1 (3.23)	-
**Age group**,** n (%)**									0.746^a^
18–30	120 (36.59)	11 (33.33)	4 (22.22)	46 (36.80)	21 (36.21)	6 (33.33)	17 (37.78)	15 (48.39)	-
31–50	110 (33.54)	15 (45.45)	8 (44.44)	40 (32.00)	19 (32.76)	6 (33.33)	14 (31.11)	8 (25.81)	-
51–70	82 (25.00)	5 (15.15)	6 (33.33)	31 (24.80)	13 (22.41)	6 (33.33)	14 (31.11)	7 (22.58)	-
70+	16 (4.88)	2 (6.06)	0 (0.00)	8 (6.40)	5 (8.62)	0 (0.00)	0 (0.00)	1 (3.23)	-
**Education**,** n (%)**									**< 0.001** ^a^ AF, AS, FG, FH, FI, FS, GH, GS, HS, IS
Primary	29 (8.84)	2 (6.06)	2 (11.11)	8 (6.40)	2 (3.45)	3 (16.67)	2 (4.44)	10 (32.26)	-
Secondary	127 (38.72)	18 (54.55)	7 (38.89)	32 (25.60)	28 (48.28)	11 (61.11)	24 (53.33)	7 (22.58)	-
Tertiary	172 (52.44)	13 (39.39)	9 (50.00)	85 (68.00)	28 (48.28)	4 (22.22)	19 (42.22)	14 (45.16)	-
**Marital status**,** n (%)**									0.326^b^
Married / cohabiting	158 (48.17)	18 (54.55)	6 (33.33)	64 (51.20)	32 (55.17)	9 (50.00)	18 (40.00)	11 (35.48)	-
Single / divorced / widow(er) / other	170 (51.83)	15 (45.45)	12 (66.67)	61 (48.80)	26 (44.83)	9 (50.00)	27 (60.00)	20 (64.52)	-
**EB type**,** n (%)**									0.002^a^ AH, BF, BH, BI, FG, FH, FI, GH, HI
Dystrophic	150 (45.73)	11 (33.33)	9 (50.00)	53 (42.40)	26 (44.83)	13 (72.22)	22 (48.89)	16 (51.61)	-
EB simplex	96 (29.27)	13 (39.39)	9 (50.00)	32 (25.60)	22 (37.93)	2 (11.11)	8 (17.78)	10 (32.26)	-
Junctional	51 (15.55)	5 (15.15)	0 (0.00)	21 (16.80)	9 (15.52)	0 (0.00)	12 (26.67)	4 (12.90)	-
Kindler	4 (1.22)	1 (3.03)	0 (0.00)	0 (0.00)	0 (0.00)	1 (5.56)	2 (4.44)	0 (0.00)	-
Don’t know	27 (8.23)	3 (9.09)	0 (0.00)	19 (15.20)	1 (1.72)	2 (11.11)	1 (2.22)	1 (3.23)	-
EB subtype*,** n (%)**									< 0.001^a^ AF, AI, FG, FH, GI
Generalized	149 (50.17)	9 (31.03)	7 (38.89)	69 (65.09)	21 (36.84)	5 (33.33)	23 (54.76)	15 (50.00)	-
Localized	81 (27.27)	9 (31.03)	7 (38.89)	27 (25.47)	12 (21.05)	4 (26.67)	13 (30.95)	9 (30.00)	-
Don’t know	67 (22.56)	11 (37.93)	4 (22.22)	10 (9.43)	24 (42.11)	6 (40.00)	6 (14.29)	6 (20.00)	-
Unknown	31	4	0	19	1	3	3	1	-
**Most frequent symptoms**,** n (%)**									
Skin blisters	313 (95.43)	32 (96.97)	18 (100.00)	119 (95.20)	57 (98.28)	17 (94.44)	43 (95.56)	27 (87.10)	0.377^a^
Pain	251 (76.52)	31 (93.94)	15 (83.33)	97 (77.60)	45 (77.59)	13 (72.22)	28 (62.22)	22 (70.97)	0.048^a^ AI, AS
Skin crusts	214 (65.24)	21 (63.64)	11 (61.11)	74 (59.20)	40 (68.97)	12 (66.67)	31 (68.89)	25 (80.65)	0.416^b^
**Severity**,** n (%)**									0.968^b^
Non-severe	137 (41.77)	12 (36.36)	8 (44.44)	50 (40.00)	27 (46.55)	7 (38.89)	19 (42.22)	14 (45.16)	-
Severe	191 (58.23)	21 (63.64)	10 (55.56)	75 (60.00)	31 (53.45)	11 (61.11)	26 (57.78)	17 (54.84)	-
**History of EB in the family**,** n (%)**	117 (35.67)	10 (30.30)	11 (61.11)	46 (36.80)	26 (44.83)	8 (44.44)	9 (20.00)	7 (22.58)	**0.017** ^a^ BI, BS, GI
**Has a caregiver**,** n (%)**	117 (35.67)	13 (39.39)	7 (38.89)	28 (22.40)	19 (32.76)	8 (44.44)	26 (57.78)	16 (51.61)	**< 0.001** ^b^ FI, FS, GI
**Principal caregiver**,** n (%)**									**0.028** ^a^ AF, FG, FI, FS
Other family member/another person	36 (30.77)	4 (30.77)	3 (42.86)	10 (35.71)	8 (42.11)	4 (50.00)	4 (15.38)	3 (18.75)	-
Parent	70 (59.83)	9 (69.23)	4 (57.14)	10 (35.71)	11 (57.89)	4 (50.00)	20 (76.92)	12 (75.00)	-
Professional carer	11 (9.40)	0 (0.00)	0 (0.00)	8 (28.57)	0 (0.00)	0 (0.00)	2 (7.69)	1 (6.25)	-
**Disability allowance**,** n (%)**	123 (37.50)	16 (48.48)	12 (66.67)	29 (23.20)	15 (25.86)	6 (33.33)	24 (53.33)	21 (67.74)	**< 0.001** ^b^ AF, AG, BF, BG, FI, FS, GI, GS, HS
**Associated in a patient-advocacy organization**,** n (%)**	173 (52.74)	29 (87.88)	8 (44.44)	57 (45.60)	33 (56.90)	4 (22.22)	16 (35.56)	26 (83.87)	**< 0.001** ^b^ AB, AF, AG, AH, AI, BS, FS, GH, GS, HS, IS

SD: Standard deviation; ^a^ Fisher’s Exact Test; ^b^ Pearson’s Chi-squared test; ^c^ Kruskal-Wallis rank sum test; Statistically significant differences between countries, based on multiple comparisons, are indicated using two-letter codes representing each country’s initials (e.g. AB means significant differences between Austria and Bulgaria)

*Kindler EB is not classified in localized or generalized, patients with Kindler EB reported unknown subtype

About one third of the patients had a caregiver to help them with the basic and instrumental need of daily living, and this was mostly done by informal caregivers (parent or another unpaid person in 90% of cases); there were differences between countries in the proportion of those who have a professional carer (reported only in France, Italy and Spain). Country-related differences were also observed in having disability allowance (more frequent in Bulgaria, Italy and Spain) and being associated in a patient-advocacy organization (more frequent in Austria and Spain) (Table 1).

### EQ-5D-5L dimensions

**Fig. 1:**
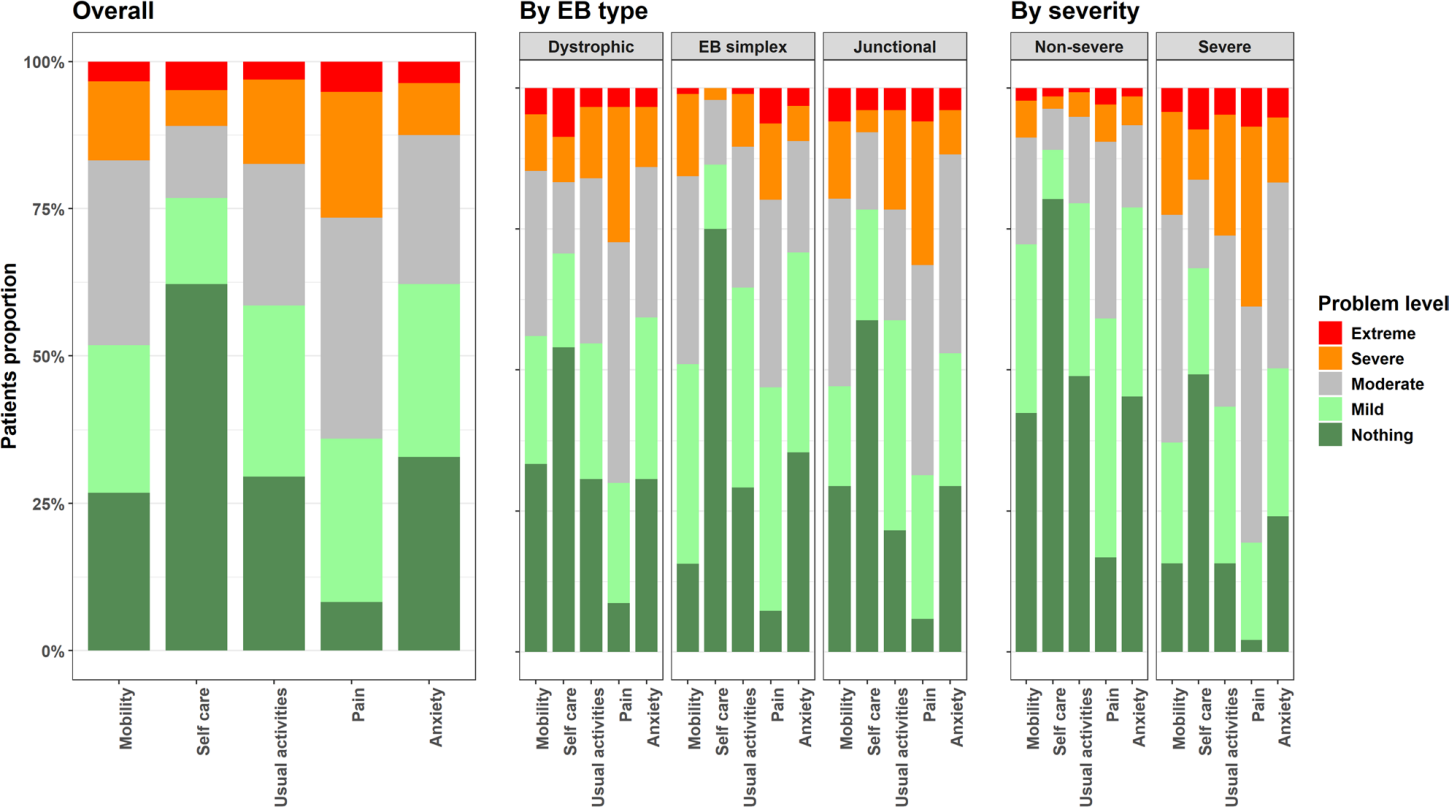
Level of problems (no problems, mild problems, moderate problems, severe problems, extreme problems) per each dimension (mobility, self-care, usual activities, pain/discomfort, anxiety/depression) for the total sample, by EB type and by severity

Of the five dimensions of EQ-5D-5L, the most affected one by EB was “pain/discomfort”: 92% of patients reported a certain level of problems in this dimension, and 27% reported severe or extreme pain/discomfort. This dimension was affected in all EB types, without statistically significant differences between them. Similarly, 12.5% of patients reported severe or extreme problems with “anxiety/depression”, with no statistically significant differences between EB types. There were statistically significant differences by severity groups in all five dimensions (more problems reported by severe cases) (*p* < 0.001). The least affected dimension was “self-care”, with 62% of patients reporting no problems, which was even higher for patients with EB simplex (75%) and non-severe level of severity (80%) (Fig. 1). Distribution of level of problems by country, EB type and severity can be found in the Supplementary material (Tables S2A-C).

### EQ-5D-5L health scores

EQ-5D value (health utility) of the overall EB sample was 0.63 (SD 0.32), ranging from 0.57 in Spain to 0.71 in Bulgaria. No statistically significant differences (*p* = 0.314) were observed between EB types: EB simplex (0.69), dystrophic EB (0.61), junctional EB (0.60) and Kindler EB (0.71). Health utility in severe cases was statistically significantly lower than in non-severe cases (0.52 vs. 0.78; *p* < 0.001) (Table 2).

**Table 2:** Mean EQ-5D-5L scores by country, EB type and severity

Variable	Overall *n* = 328	Austria *n* = 33	Bulgaria *n* = 18	France *n* = 125	Germany *n* = 58	Hungary *n* = 18	Italy *n* = 45	Spain *n* = 31
**EQ-5D value, mean (SD)**								
**EB type (** ***p*** **-value for EQ-5D value by EB type = 0.314** ^**a**^ **)**								
Dystrophic	0.61 (0.32)	0.51 (0.45)	0.75 (0.32)	0.72 (0.23)	0.61 (0.38)	0.57 (0.43)	0.55 (0.39)	0.58 (0.23)
EB simplex	0.69 (0.28)	0.82 (0.13)	0.67 (0.20)	0.72 (0.30)	0.71 (0.35)	0.90 (0.02)	0.70 (0.21)	0.63 (0.27)
Junctional	0.60 (0.32)	0.80 (0.18)	-	0.57 (0.35)	0.74 (0.25)	-	0.61 (0.34)	0.48 (0.22)
Kindler	0.71 (0.27)	0.90 (NA)	-	-	-	0.23 (NA)	0.81 (0.15)	-
** Severity (p-value for EQ-5D value by severity < 0.001** ^**a**^ **)**								
Non-severe	0.78 (0.23)	0.86 (0.09)	0.78 (0.21)	0.83 (0.22)	0.87 (0.12)	0.80 (0.32)	0.72 (0.26)	0.63 (0.27)
Severe	0.52 (0.34)	0.53 (0.47)	0.66 (0.30)	0.62 (0.28)	0.47 (0.40)	0.45 (0.42)	0.51 (0.38)	0.52 (0.21)
** Total sample**	**0.63 (0.32)**	**0.65 (0.41)**	**0.71 (0.26)**	**0.70 (0.28)**	**0.66 (0.36)**	**0.59 (0.41)**	**0.60 (0.35)**	**0.57 (0.24)**
**EQ VAS**,** mean (SD)**^**b**^								
**EB type (p-value for EQ VAS by EB type = 0.011** ^**a, c**^ **)**								
Dystrophic	56.50 (23.61)	51.45 (30.66)	63.00 (24.04)	57.42 (22.26)	57.24 (24.33)	49.00 (23.09)	59.77 (27.48)	53.75 (17.22)
EB simplex	67.59 (19.47)	75.62 (17.46)	66.43 (11.44)	66.28 (21.48)	68.36 (19.97)	83.00 (11.31)	59.75 (17.21)	63.60 (20.77)
Junctional	57.88 (22.21)	68.00 (19.24)	-	52.48 (21.03)	66.33 (30.31)	-	59.00 (20.23)	51.25 (13.30)
Kindler	60.50 (17.54)	80.00 (NA)	-	-	-	42.00 (NA)	60.00 (14.14)	-
** Severity (p-value for EQ VAS by severity < 0.001** ^**a**^ **)**								
Non-severe	69.82 (20.10)	74.83 (17.87)	69.86 (16.79)	72.18 (19.93)	74.35 (18.46)	66.29 (23.99)	64.21 (21.85)	58.07 (19.97)
Severe	53.04 (22.68)	57.00 (31.02)	60.33 (20.64)	51.95 (20.87)	52.71 (23.89)	41.91 (19.36)	55.12 (23.56)	53.76 (18.00)
** Total sample**	**60.01 (23.15)**	**63.48 (28.06)**	**64.50 (19.07)**	**60.04 (22.72)**	**62.58 (24.00)**	**51.39 (23.94)**	**58.96 (23.05)**	**55.71 (18.72)**

^a^ Significance within each category (EB type, severity level) using Kruskal-Wallis rank sum test; ^b^ Total sample for EQ VAS: *n* = 325 (Bulgaria *n* = 16; Germany *n* = 57); ^c^ Statistically significant difference between dystrophic and EB simplex

EQ-5D-5L country-specific value sets used for France (Andrade et al., 2020), Germany (Ludwig et al., 2018), Hungary (Rencz et al., 2020), Italy (Finch et al., 2022) and Spain (Ramos-Goñi et al., 2018); EQ-5D-5L regional value sets (Łaszewska et al., 2022) used for Austria – Central Western value set, Bulgaria – Eastern European value set and Overall sample – Central Western value set

The average EQ VAS of the overall EB sample was 60 (SD 23.15), ranging from 51.4 in Hungary to 64.5 in Bulgaria. Similar to EQ-5D value, patients with EB simplex reported higher EQ VAS (67.6) than patients with dystrophic EB (56.5), junctional EB (57.9) and Kindler EB (60.5) (statistical significance between EB simplex and dystrophic; *p* = 0.011). Also, severity was related to statistically significantly lower EQ VAS (53 in severe cases vs. 69.8 in non-severe cases; *p* < 0.001) (Table 2).

There were no statistically significant differences between countries in all subgroups, except for health utilities in non-severe cases and total EB sample, which can be due to country-specific value sets used. When supra-national value sets (i.e. Central Western, Eastern European and Southern value set) were used [19], these differences were no more detectable (see Table S3 in Supplementary Material). In 14.33% of cases the questionnaire was completed by a caregiver on behalf of the person with EB (proxy version of EQ-5D-5L), and these responses did not differ significantly from those completed by patients themselves (p-value > 0.9 for EQ-5D value).

To quantify the impact of EB on health utilities, we considered general population norms in each country, i.e. health utilities and EQ VAS measured in a representative sample of general population in each country. Comparing these values with the values of the EB population in this study, it was observed that EB patients in all countries had statistically significantly lower values than the general population in their country (*p* ≤ 0.005). The effect sizes, calculated with Cohen’s *d*, reveal in all countries very pronounced difference (substantial effect) in EQ-5D value (*d* ≥ 0.8), and medium to substantial effect in EQ VAS (*d* ≥ 0.5), with EB patients reporting much poorer HRQoL than general populations (Table 3).

**Table 3:** Differences in EQ-5D scores between EB population and general population by country

	EQ-5D value				EQ VAS					
	EB patients mean (SD)	Population norms^a^ mean (SD)	t-student (*p*-value)	Effect size^b^	EB patients mean (SD)		Population norms^a^ mean (SD)	t-student (*p*-value)	Effect size^b^	
Austria	0.646 (0.412)	0.861 (0.189)^c^	0.005	1.077	63.48 (28.06)		76.80 (18.50)^c^	0.011	0.706	
Bulgaria	0.713 (0.263)	0.942 (0.127)	0.002	1.756	64.50 (19.07)		77.90 (19.00)	0.009	0.705	
France	0.701 (0.279)	0.905 (0.158)	< 0.001	1.280	60.04 (22.72)		73.44 (22.20)	< 0.001	0.603	
Germany	0.657 (0.360)	0.861 (0.189)	< 0.001	1.010	62.58 (24.00)		76.80 (18.50)	< 0.001	0.755	
Hungary	0.589 (0.413)	0.870 (0.210)	0.010	1.334	51.39 (23.94)		75.22 (19.91)	0.001	1.196	
Italy	0.596 (0.346)	0.893 (0.131)	< 0.001	2.026	58.96 (23.05)		76.10 (15.90)	< 0.001	1.054	
Spain	0.572 (0.243)	0.865 (0.171)	< 0.001	1.689	55.71 (18.72)		76.90 (16.40)	< 0.001	1.286	

^a^ EQ-5D-5L population norms for each country (Dewilde et al., 2025; Encheva et al., 2020; Gautier et al., 2023; Pentek et al., 2025); ^b^ Effect size calculated using Cohen’s *d*. Small but meaningful effect: *d* ≈ 0.2; Moderate effect: *d* ≈ 0.5; Large effect: *d* ≈ 0.8; ^c^ Population norms of Germany used for Austria

### Determinants of EQ-5D-5L results

The univariate analysis shows that many symptoms and complications, and some sociodemographic variables, are individually associated with HRQoL in EB patients. In the multivariate model, which accounts for confounding and overlapping effects, among the symptoms, only pain, chronic wounds, joint deformities, functional disability, and wheelchair use remained independently associated with EQ-5D value, with wheelchair use showing the largest negative effect. The final model explained 39% of the variance in EQ-5D value (R² = 0.387) (Table S4A in the Supplementary material). EQ VAS values were independently associated only with chronic wounds and functional disability and the model could explain 25% of the variance in EQ VAS (R² = 0.251) (Table S4B in the Supplementary material).

## Discussion

The accurate measurement and valuation of HRQoL are crucial for understanding the full impact of a disease and for informing healthcare resource allocation decisions, particularly through the use of utility values derived from generic instruments like the EQ-5D-5L. Despite the profound and lifelong burden of EB on patients and their caregivers, there remains a notable scarcity of comprehensive and consistently reported utility data for EB patient populations [25]. This lack of detailed utility evidence poses a challenge for robust economic assessments and comparative analyses of the disease’s impact. The present study addresses this gap by generating new utility data using the EQ-5D-5L in a large multinational sample of adult EB patients, thereby contributing a substantial addition to the evidence base. This is particularly relevant considering that three treatments for dystrophic EB wounds – a topical gel containing a birch bark extract (Oleogel-S10), a topical gene-therapy based gel (beremagene geperpavec), and a cell sheet-based gene therapy (prademagene zamikeracel) – have been recently approved by U.S. Food and Drug Administration and the first two by European Medicine Agency [26–28].

While the generalizability and comparability across different diseases are significant advantages of generic instruments, it is crucial to understand their performance in specific patient populations. A recent comparative study by Szabó et al. (2025) focused on the measurement properties of various generic utility assessment methods, including the EQ-5D-5L, PROPr, SF-6D, and Time Trade-Off, in patients with chronic skin diseases [29]. The EQ-5D-5L was found to be the most efficient in discriminating between known patient groups in terms of physical health, while also benefiting from the widest availability of national value sets and a short, practical format. These attributes support its feasibility for routine use in EB, where the disease burden spans multiple HRQoL domains and where consistent, standardised reporting is critical for international comparability.

Previous international and national efforts have attempted to quantify the HRQoL and socioeconomic burden of EB. The BURQOL-RD initiative (2010–2013), which included eight European countries, applied EQ-5D-3 L in 111 adult EB patients and reported mean EQ-5D value scores of 0.579, ranging from 0.49 in Hungary to 0.71 in Sweden [12]. A later multi-country study focusing on 46 adults with dystrophic EB reported even lower mean EQ-5D value scores (0.456) [30]. Similarly, a national Italian study assessed HRQoL in 125 EB patients using the EQ-5D-5L but did not present derived utility scores, instead reported descriptive system and EQ VAS outcomes (mean ± SD of 62 ± 23 for adults, 59 ± 19 for children) [11]. A more recent Spanish study provided utility values for 39 patients, with mean scores of 0.45 for dystrophic, junctional, and Kindler EB, and 0.62 for EB simplex [9].

In our study of 328 adult EB patients across seven European countries, the mean EQ-5D-5L utility value was 0.63 (SD 0.32), ranging from 0.57 in Spain to 0.71 in Bulgaria, and the mean EQ VAS was 60 (SD 23.15), without significant differences between EB types. The differences between our findings and previous results—such as those from [9] or [12, 30]—may be explained by differences in patient cohorts, inclusion criteria, the EQ-5D version used, and the value sets applied. For example, earlier studies often used the EQ-5D-3L, included paediatric participants, or relied on older national EQ-5D value sets. Advances in supportive care may also have contributed to better HRQoL outcomes in our cohort.

Validated EB severity scores such as the Epidermolysis Bullosa Disease Activity and Scarring Index (EBDASI) or the Instrument for Scoring Clinical Outcomes of Research for Epidermolysis Bullosa (iscorEB) are available [31]. However, they need to be filled by healthcare professionals and could not be used in our patient self-reported survey. Thus, it became necessary to establish an internal rule for distinguishing between non-severe and severe level of the condition for the purposes of our analysis. This approach identified “severe” EB as the presence of pain plus at least one of seven additional severe symptoms (chronic wounds, malnutrition, joint deformities, functional disabilities, squamous cell carcinoma, gastrostomy, or narrowed oesophagus). It is crucial to emphasize that this symptom-based stratification was developed strictly for the internal purposes of our study analysis and is not intended to be a new clinical severity scale; rather, it allowed for a more granular understanding of disease burden within our dataset. We found this severity evaluation essential because merely categorizing health utilities by EB type alone, as seen in some other HRQoL research, may not adequately capture the wide spectrum of clinical severity. For example, our analysis revealed a statistically significant difference in health utility between our defined “severe” cases (0.52) and “non-severe” cases (0.78). This contrasts with the approach taken by [9], who broadly classified “severe EB” to include all dystrophic, junctional, and Kindler EB patients, while “non-severe EB” was limited to simplex EB, reporting mean utility values of 0.45 and 0.62 respectively [9]. We believe this major EB type-based classification may not fully reflect the individual patient’s severity (as simplex, junctional and dystrophic EB major types each comprise several subtypes of markedly different severity), which was confirmed by our findings.

Across all available studies, including ours, EB patients consistently report HRQoL far below general population levels. Standardized effect size methodology, such as Cohen’s *d*, have been applied in various studies, providing a meaningful way to contextualize the health burden of a disease [32–34]. By comparing EQ-5D outcomes of adults with EB to country-specific general population norms, we observed exceptionally large differences, well above conventional thresholds for “large” effects. A limitation of this comparison is that the demographic profile of the study cohort does not perfectly reflect the general adult population of the participating countries. Our sample included a higher proportion of women (61% vs. approx. 50% in the EU) and was younger overall. Because HRQoL in the general population typically declines with advancing age, the younger age distribution would normally be expected to yield higher EQ-5D utilities, whereas the female predominance might have a small downward effect. Importantly, while effect size benchmarks must be interpreted carefully in rare diseases, where even smaller differences can be clinically meaningful [33], our findings highlight that EB imposes a burden far greater than what is typically considered substantial in population health research. It is also important to note that population norms themselves can change over time and may vary depending on the specific country’s value set used, as demonstrated by how our study’s observed country-specific differences in health utilities for the total EB sample were no more detected when supra-national value sets were applied [19].

Our analysis shows that although many clinical features of EB were individually associated with reduced HRQoL, only a limited set of factors emerged as independent determinants when accounting for confounding effects. This highlights that both symptomatic burden and functional impairments are the primary drivers of HRQoL in EB. Among these, wheelchair use had the strongest impact, revealing the profound consequences of loss of mobility on patient-perceived health. These findings emphasize the need for comprehensive symptom management and interventions that preserve function and independence in EB care. From an economic evaluation perspective, they also suggest that targeting these domains may yield the greatest improvements in health utility and cost-effectiveness of interventions.

### Limitations

Several limitations should be considered when interpreting these results. First, the cross-sectional design captures HRQoL at a single point in time and does not reflect changes over the disease course or in response to treatment. Second, despite being one of the largest multinational EB cohorts studied to date, the sample remains small in absolute terms due to the rarity of the condition and may not be fully representative of the broader EB population in each country. Third, the recruitment strategy, which relied in part on patient organisations and self-selection, may have led to participation bias. Fourth, HRQoL outcomes were self-reported and may be subject to recall bias or individual interpretation of questions; besides, a small part of the questionnaires was completed by caregivers on behalf of the patients. Fifth, while our symptom-based severity classification was developed with clinical expert input, it has not been validated against established scales such as the EBDASI or the iscorEB [31]. Finally, our study focused exclusively on adult patients, limiting the applicability of the findings to paediatric populations. Future research should aim to address these limitations through longitudinal, population-based, and mixed-method designs.

## Conclusions

This study provides the most extensive and up-to-date utility data for EB patients across multiple European countries, offering a robust, patient-centred quantification of the disease’s impact on health-related quality of life. These results fill a critical evidence gap, supporting integration of EB-specific data into health economic models and policy discussions. Importantly, the availability of standardized utility values will facilitate direct comparisons of EB with other chronic and rare conditions, thereby enhancing the visibility of EB in broader health prioritization frameworks and resource allocation.

## Supplementary Information

Below is the link to the electronic supplementary material.

Supplementary Material 1

## Data Availability

The datasets used and/or analysed during the current study are available from the corresponding author on reasonable request.

## Abbreviations

EB: Epidermolysis bullosa
HRQoL: Health-related quality of life
EQ-5D: EuroQol five dimensions questionnaire
EQ-5D-5L: EuroQol five dimensions questionnaire with five response levels
EQ-5D-3L: EuroQol five dimensions questionnaire with three response levels
EQ VAS: Visual analogue scale, part of the EQ-5D questionnaire
SD: Standard deviation
EBDASI: Epidermolysis bullosa disease activity and scarring index
iscorEB: Instrument for scoring clinical outcomes of research for epidermolysis bullosa
PAO: Patient advocacy organization
