# Supplementary material for: Health-related quality of life in adults with epidermolysis bullosa: a cross-sectional study in seven European countries using EQ-5D-5L
Source: Orphanet J Rare Dis. 2026 Mar 19;21:167. doi: 10.1186/s13023-026-04299-1 (PMC13122926; doi:10.1186/s13023-026-04299-1)
Supplement: Supplementary file 1 — Supplementary Material 1 [file 13023_2026_4299_MOESM1_ESM.pdf]

Supplementary Material

Health-related quality of life in adults with epidermolysis bullosa: a cross-sectional study in seven European countries using EQ-5D-5L

Table S1A. Clinical characteristics by country

Variable	Overall n = 328	Austria n = 33	Bulgaria n = 18	France n = 125	Germany n = 58	Hungary n = 18	Italy n = 45	Spain n = 31	p-value
**Symptoms, n (%)**									
Skin blisters	313 (95.43)	32 (96.97)	18 (100.00)	119 (95.20)	57 (98.28)	17 (94.44)	43 (95.56)	27 (87.10)	0.377[a]
Pain	251 (76.52)	31 (93.94)	15 (83.33)	97 (77.60)	45 (77.59)	13 (72.22)	28 (62.22)	22 (70.97)	**0.048**[a]<br>AI,AS
Skin crusts	214 (65.24)	21 (63.64)	11 (61.11)	74 (59.20)	40 (68.97)	12 (66.67)	31 (68.89)	25 (80.65)	0.416[b]
Pruritus	210 (64.02)	30 (90.91)	13 (72.22)	52 (41.60)	42 (72.41)	15 (83.33)	36 (80.00)	22 (70.97)	**<0.001**[b]<br>AF,FG,FH,FI,FS
Atrophic scars	201 (61.28)	17 (51.52)	11 (61.11)	83 (66.40)	33 (56.90)	11 (61.11)	25 (55.56)	21 (67.74)	0.630[b]
Mucosal involvement	166 (50.61)	16 (48.48)	6 (33.33)	60 (48.00)	34 (58.62)	9 (50.00)	25 (55.56)	16 (51.61)	0.610[b]
Functional disability	158 (48.17)	15 (45.45)	9 (50.00)	55 (44.00)	26 (44.83)	8 (44.44)	27 (60.00)	18 (58.06)	0.532[b]
Chronic wounds	148 (45.12)	15 (45.45)	7 (38.89)	60 (48.00)	21 (36.21)	8 (44.44)	20 (44.44)	17 (54.84)	0.709[b]
Join deformities	97 (29.57)	8 (24.24)	9 (50.00)	33 (26.40)	11 (18.97)	8 (44.44)	15 (33.33)	13 (41.94)	0.056[b]
Teeth loss	97 (29.57)	11 (33.33)	5 (27.78)	33 (26.40)	17 (29.31)	5 (27.78)	18 (40.00)	8 (25.81)	0.751[b]
Malnutrition	78 (23.78)	9 (27.27)	3 (16.67)	25 (20.00)	11 (18.97)	5 (27.78)	18 (40.00)	7 (22.58)	0.186[a]
Esophageal dilatations	70 (21.34)	8 (24.24)	0 (0.00)	22 (17.60)	15 (25.86)	1 (5.56)	17 (37.78)	7 (22.58)	**0.011**[a]<br>AB,BG,BI,BS,FI,HI
Wheelchair	45 (13.72)	4 (12.12)	1 (5.56)	14 (11.20)	9 (15.52)	3 (16.67)	10 (22.22)	4 (12.90)	0.589[a]
Squamous cell carcinoma	44 (13.41)	2 (6.06)	3 (16.67)	15 (12.00)	4 (6.90)	3 (16.67)	10 (22.22)	7 (22.58)	0.129[a]
Gastrostomy	23 (7.01)	1 (3.03)	2 (11.11)	7 (5.60)	6 (10.34)	1 (5.56)	4 (8.89)	2 (6.45)	0.767[a]
**EB type and subtype, n(%)**									**<0.001**[a]<br>AF,BF,FG,FH,FI,GI,HI
Dystrophic Generalized	83 (25.30)	4 (12.12)	5 (27.78)	35 (28.00)	13 (22.41)	5 (27.78)	12 (26.67)	9 (29.03)	
Dystrophic Localized	37 (11.28)	2 (6.06)	2 (11.11)	14 (11.20)	4 (6.90)	4 (22.22)	6 (13.33)	5 (16.13)	
Dystrophic subtype NR	30 (9.15)	5 (15.15)	2 (11.11)	4 (3.20)	9 (15.52)	4 (22.22)	4 (8.89)	2 (6.45)	
EB simplex Generalized	33 (10.06)	3 (9.09)	2 (11.11)	19 (15.20)	3 (5.17)	0 (0.00)	2 (4.44)	4 (12.90)	
EB simplex Localized	39 (11.89)	7 (21.21)	5 (27.78)	11 (8.80)	7 (12.07)	0 (0.00)	6 (13.33)	3 (9.68)	
EB simplex subtype NR	24 (7.32)	3 (9.09)	2 (11.11)	2 (1.60)	12 (20.69)	2 (11.11)	0 (0.00)	3 (9.68)	

Variable	Overall n = 328	Austria n = 33	Bulgaria n = 18	France n = 125	Germany n = 58	Hungary n = 18	Italy n = 45	Spain n = 31	p-value
Junctional Generalized	33 (10.06)	2 (6.06)	0 (0.00)	15 (12.00)	5 (8.62)	0 (0.00)	9 (20.00)	2 (6.45)	
Junctional Localized	5 (1.52)	0 (0.00)	0 (0.00)	2 (1.60)	1 (1.72)	0 (0.00)	1 (2.22)	1 (3.23)	
Junctional subtype NR	13 (3.96)	3 (9.09)	0 (0.00)	4 (3.20)	3 (5.17)	0 (0.00)	2 (4.44)	1 (3.23)	
Kindler subtype NR	4 (1.22)	1 (3.03)	0 (0.00)	0 (0.00)	0 (0.00)	1 (5.56)	2 (4.44)	0 (0.00)	
EB type and subtype NR	27 (8.23)	3 (9.09)	0 (0.00)	19 (15.20)	1 (1.72)	2 (11.11)	1 (2.22)	1 (3.23)	

[a] Fisher's Exact Test; [b] Pearson's Chi-squared test; Statistically significant differences between countries, based on multiple comparisons, are indicated using two-letter codes representing each country's initials (e.g. AB means significant differences between Austria and Bulgaria); NR=Not reported.

Table S1B. Clinical characteristics by severity

Variable	Overall<br>n = 328	Non-severe<br>n = 137	Severe<br>n = 191	p-value
**Symptoms, n (%)**				
Skin blisters	313 (95.43)	128 (93.43)	185 (96.86)	0.143[a]
Pain	251 (76.52)	60 (43.80)	191 (100.00)	<0.001[a]
Skin crusts	214 (65.24)	70 (51.09)	144 (75.39)	<0.001[a]
Pruritus	210 (64.02)	68 (49.64)	142 (74.35)	<0.001[a]
Atrophic scars	201 (61.28)	64 (46.72)	137 (71.73)	<0.001[a]
Mucosal involvement	166 (50.61)	43 (31.39)	123 (64.40)	<0.001[a]
Functional disability	158 (48.17)	21 (15.33)	137 (71.73)	<0.001[a]
Chronic wounds	148 (45.12)	16 (11.68)	132 (69.11)	<0.001[a]
Join deformities	97 (29.57)	15 (10.95)	82 (42.93)	<0.001[a]
Teeth loss	97 (29.57)	21 (15.33)	76 (39.79)	<0.001[a]
Malnutrition	78 (23.78)	10 (7.30)	68 (35.60)	<0.001[a]
Esophageal dilatations	70 (21.34)	12 (8.76)	58 (30.37)	<0.001[a]
Wheelchair	45 (13.72)	6 (4.38)	39 (20.42)	<0.001[a]
Squamous cell carcinoma	44 (13.41)	9 (6.57)	35 (18.32)	0.002[a]
Gastrostomy	23 (7.01)	1 (0.73)	22 (11.52)	<0.001[a]
**EB type and subtype, n (%)**				0.001[b]
Dystrophic Generalized	83 (25.30)	18 (13.14)	65 (34.03)	
Dystrophic Localized	37 (11.28)	14 (10.22)	23 (12.04)	
Dystrophic subtype NR	30 (9.15)	15 (10.95)	15 (7.85)	
EB simplex Generalized	33 (10.06)	17 (12.41)	16 (8.38)	
EB simplex Localized	39 (11.89)	20 (14.60)	19 (9.95)	
EB simplex subtype NR	24 (7.32)	16 (11.68)	8 (4.19)	
Junctional Generalized	33 (10.06)	12 (8.76)	21 (10.99)	
Junctional Localized	5 (1.52)	3 (2.19)	2 (1.05)	
Junctional subtype NR	13 (3.96)	4 (2.92)	9 (4.71)	
Kindler subtype NR	4 (1.22)	3 (2.19)	1 (0.52)	
EB type and subtype NR	27 (8.23)	15 (10.95)	12 (6.28)	
**Sex, n (%)**				0.217[b]
Female	200 (60.98)	88 (64.23)	112 (58.64)	
Male	127 (38.72)	48 (35.04)	79 (41.36)	
Other	1 (0.30)	1 (0.73)	0 (0.00)	
**Age group, n (%)**				0.968[a]
18-30	120 (36.59)	52 (37.96)	68 (35.60)	
31-50	110 (33.54)	45 (32.85)	65 (34.03)	

Variable	Overall<br>n = 328	Non-severe<br>n = 137	Severe<br>n = 191	p-value
51-70	82 (25.00)	33 (24.09)	49 (25.65)	
70+	16 (4.88)	7 (5.11)	9 (4.71)	
**Has a caregiver, n (%)**	117 (35.67)	30 (21.90)	87 (45.55)	<0.001[a]
**Disability allowance, n (%)**	123 (37.50)	38 (27.74)	85 (44.50)	0.002[a]
**Associated in a patient-advocacy organization, n (%)**	173 (52.74)	63 (45.99)	110 (57.59)	0.038[a]

[a] Pearson's Chi-squared test; [b] Fisher's Exact Test

Table S2A. Distribution of level of problems by country

Variable	Overall n = 328	Austria n = 33	Bulgaria n = 18	France n = 125	Germany n = 58	Hungary n = 18	Italy n = 45	Spain n = 31	p-value
**Mobility, n (%)**									0.071[a]
No problems	88 (26.83)	10 (30.30)	4 (22.22)	25 (20.00)	25 (43.10)	4 (22.22)	16 (35.56)	4 (12.90)	
Mild problems	82 (25.00)	10 (30.30)	6 (33.33)	32 (25.60)	13 (22.41)	4 (22.22)	8 (17.78)	9 (29.03)	
Moderate problems	103 (31.40)	6 (18.18)	4 (22.22)	46 (36.80)	10 (17.24)	6 (33.33)	15 (33.33)	16 (51.61)	
Severe problems	44 (13.41)	7 (21.21)	3 (16.67)	16 (12.80)	9 (15.52)	3 (16.67)	4 (8.89)	2 (6.45)	
Extreme problems	11 (3.35)	0 (0.00)	1 (5.56)	6 (4.80)	1 (1.72)	1 (5.56)	2 (4.44)	0 (0.00)	
**Self-care, n (%)**									0.007[a]
No problems	204 (62.20)	24 (72.73)	10 (55.56)	87 (69.60)	42 (72.41)	10 (55.56)	18 (40.00)	13 (41.94)	
Mild problems	48 (14.63)	2 (6.06)	4 (22.22)	16 (12.80)	6 (10.34)	3 (16.67)	9 (20.00)	8 (25.81)	
Moderate problems	40 (12.20)	1 (3.03)	3 (16.67)	14 (11.20)	3 (5.17)	3 (16.67)	10 (22.22)	6 (19.35)	
Severe problems	20 (6.10)	2 (6.06)	1 (5.56)	5 (4.00)	6 (10.34)	0 (0.00)	5 (11.11)	1 (3.23)	
Extreme problems	16 (4.88)	4 (12.12)	0 (0.00)	3 (2.40)	1 (1.72)	2 (11.11)	3 (6.67)	3 (9.68)	
**Usual activities, n (%)**									0.106[a]
No problems	97 (29.57)	13 (39.39)	3 (16.67)	37 (29.60)	26 (44.83)	5 (27.78)	8 (17.78)	5 (16.13)	
Mild problems	95 (28.96)	10 (30.30)	8 (44.44)	33 (26.40)	12 (20.69)	5 (27.78)	17 (37.78)	10 (32.26)	
Moderate problems	79 (24.09)	4 (12.12)	5 (27.78)	36 (28.80)	9 (15.52)	4 (22.22)	10 (22.22)	11 (35.48)	
Severe problems	47 (14.33)	3 (9.09)	2 (11.11)	17 (13.60)	10 (17.24)	3 (16.67)	9 (20.00)	3 (9.68)	
Extreme problems	10 (3.05)	3 (9.09)	0 (0.00)	2 (1.60)	1 (1.72)	1 (5.56)	1 (2.22)	2 (6.45)	
**Pain/discomfort, n (%)**									0.193[a]
No problems	27 (8.23)	3 (9.09)	2 (11.11)	4 (3.20)	7 (12.07)	3 (16.67)	5 (11.11)	3 (9.68)	
Mild problems	91 (27.74)	14 (42.42)	5 (27.78)	30 (24.00)	17 (29.31)	6 (33.33)	11 (24.44)	8 (25.81)	
Moderate problems	123 (37.50)	11 (33.33)	8 (44.44)	51 (40.80)	18 (31.03)	5 (27.78)	21 (46.67)	9 (29.03)	
Severe problems	70 (21.34)	3 (9.09)	2 (11.11)	36 (28.80)	12 (20.69)	3 (16.67)	5 (11.11)	9 (29.03)	
Extreme problems	17 (5.18)	2 (6.06)	1 (5.56)	4 (3.20)	4 (6.90)	1 (5.56)	3 (6.67)	2 (6.45)	
**Anxiety/depression, n (%)**									0.069[a]
No problems	108 (32.93)	15 (45.45)	3 (16.67)	39 (31.20)	15 (25.86)	8 (44.44)	14 (31.11)	14 (45.16)	
Mild problems	96 (29.27)	8 (24.24)	8 (44.44)	35 (28.00)	19 (32.76)	5 (27.78)	15 (33.33)	6 (19.35)	
Moderate problems	83 (25.30)	4 (12.12)	7 (38.89)	33 (26.40)	19 (32.76)	3 (16.67)	12 (26.67)	5 (16.13)	
Severe problems	29 (8.84)	1 (3.03)	0 (0.00)	12 (9.60)	5 (8.62)	2 (11.11)	3 (6.67)	6 (19.35)	
Extreme problems	12 (3.66)	5 (15.15)	0 (0.00)	6 (4.80)	0 (0.00)	0 (0.00)	1 (2.22)	0 (0.00)	

[a] Fisher's Exact Test

Table S2B. Distribution of level of problems by severity

Variable	Overall<br>n = 328	Non-severe<br>n = 137	Severe<br>n = 191	p-value
**Mobility, n (%)**				<0.001[a]
No problems	88 (26.83)	58 (42.34)	30 (15.71)	
Mild problems	82 (25.00)	41 (29.93)	41 (21.47)	
Moderate problems	103 (31.40)	26 (18.98)	77 (40.31)	
Severe problems	44 (13.41)	9 (6.57)	35 (18.32)	
Extreme problems	11 (3.35)	3 (2.19)	8 (4.19)	
**Self-care, n (%)**				<0.001[b]
No problems	204 (62.20)	110 (80.29)	94 (49.21)	
Mild problems	48 (14.63)	12 (8.76)	36 (18.85)	
Moderate problems	40 (12.20)	10 (7.30)	30 (15.71)	
Severe problems	20 (6.10)	3 (2.19)	17 (8.90)	
Extreme problems	16 (4.88)	2 (1.46)	14 (7.33)	
**Usual activities, n (%)**				<0.001[a]
No problems	97 (29.57)	67 (48.91)	30 (15.71)	
Mild problems	95 (28.96)	42 (30.66)	53 (27.75)	
Moderate problems	79 (24.09)	21 (15.33)	58 (30.37)	
Severe problems	47 (14.33)	6 (4.38)	41 (21.47)	
Extreme problems	10 (3.05)	1 (0.73)	9 (4.71)	
**Pain/discomfort, n (%)**				<0.001[b]
No problems	27 (8.23)	23 (16.79)	4 (2.09)	
Mild problems	91 (27.74)	58 (42.34)	33 (17.28)	
Moderate problems	123 (37.50)	43 (31.39)	80 (41.88)	
Severe problems	70 (21.34)	9 (6.57)	61 (31.94)	
Extreme problems	17 (5.18)	4 (2.92)	13 (6.81)	
**Anxiety/depression, n (%)**				<0.001[b]
No problems	108 (32.93)	62 (45.26)	46 (24.08)	
Mild problems	96 (29.27)	46 (33.58)	50 (26.18)	
Moderate problems	83 (25.30)	20 (14.60)	63 (32.98)	
Severe problems	29 (8.84)	7 (5.11)	22 (11.52)	
Extreme problems	12 (3.66)	2 (1.46)	10 (5.24)	

[a] Fisher's Exact Test; [b] Pearson's Chi-squared test

Table S2C. Distribution of level of problems by EB type

Variable	Overall<br>n = 328	Dystrophic<br>n = 150	EB simplex<br>n = 96	Junctional<br>n = 51	Kindler<br>n = 4	Don't know<br>n = 27	p-value
**Mobility, n (%)**							0.029[a]
No problems	88 (26.83)	50 (33.33)	15 (15.63)	15 (29.41)	2 (50.00)	6 (22.22)	
Mild problems	82 (25.00)	34 (22.67)	34 (35.42)	9 (17.65)	1 (25.00)	4 (14.81)	
Moderate problems	103 (31.40)	44 (29.33)	32 (33.33)	17 (33.33)	0 (0.00)	10 (37.04)	
Severe problems	44 (13.41)	15 (10.00)	14 (14.58)	7 (13.73)	1 (25.00)	7 (25.93)	
Extreme problems	11 (3.35)	7 (4.67)	1 (1.04)	3 (5.88)	0 (0.00)	0 (0.00)	
**Self-care, n (%)**							0.030[a]
No problems	204 (62.20)	81 (54.00)	72 (75.00)	30 (58.82)	4 (100.00)	17 (62.96)	
Mild problems	48 (14.63)	25 (16.67)	11 (11.46)	10 (19.61)	0 (0.00)	2 (7.41)	
Moderate problems	40 (12.20)	19 (12.67)	11 (11.46)	7 (13.73)	0 (0.00)	3 (11.11)	
Severe problems	20 (6.10)	12 (8.00)	2 (2.08)	2 (3.92)	0 (0.00)	4 (14.81)	
Extreme problems	16 (4.88)	13 (8.67)	0 (0.00)	2 (3.92)	0 (0.00)	1 (3.70)	
**Usual activities, n (%)**							0.016[a]
No problems	97 (29.57)	46 (30.67)	28 (29.17)	11 (21.57)	2 (50.00)	10 (37.04)	
Mild problems	95 (28.96)	36 (24.00)	34 (35.42)	19 (37.25)	1 (25.00)	5 (18.52)	
Moderate problems	79 (24.09)	44 (29.33)	24 (25.00)	10 (19.61)	0 (0.00)	1 (3.70)	
Severe problems	47 (14.33)	19 (12.67)	9 (9.38)	9 (17.65)	1 (25.00)	9 (33.33)	
Extreme problems	10 (3.05)	5 (3.33)	1 (1.04)	2 (3.92)	0 (0.00)	2 (7.41)	
**Pain/discomfort, n (%)**							0.134[a]
No problems	27 (8.23)	13 (8.67)	7 (7.29)	3 (5.88)	0 (0.00)	4 (14.81)	
Mild problems	91 (27.74)	32 (21.33)	38 (39.58)	13 (25.49)	2 (50.00)	6 (22.22)	
Moderate problems	123 (37.50)	64 (42.67)	32 (33.33)	19 (37.25)	1 (25.00)	7 (25.93)	
Severe problems	70 (21.34)	36 (24.00)	13 (13.54)	13 (25.49)	1 (25.00)	7 (25.93)	
Extreme problems	17 (5.18)	5 (3.33)	6 (6.25)	3 (5.88)	0 (0.00)	3 (11.11)	
**Anxiety/depression, n (%)**							0.667[a]
No problems	108 (32.93)	46 (30.67)	34 (35.42)	15 (29.41)	1 (25.00)	12 (44.44)	
Mild problems	96 (29.27)	43 (28.67)	34 (35.42)	12 (23.53)	2 (50.00)	5 (18.52)	
Moderate problems	83 (25.30)	40 (26.67)	19 (19.79)	18 (35.29)	1 (25.00)	5 (18.52)	
Severe problems	29 (8.84)	16 (10.67)	6 (6.25)	4 (7.84)	0 (0.00)	3 (11.11)	
Extreme problems	12 (3.66)	5 (3.33)	3 (3.13)	2 (3.92)	0 (0.00)	2 (7.41)	

[a] Fisher's Exact Test

Table S3. EQ-5D-5L value results with other value sets

Variable	Austria<br>n = 33	Bulgaria<br>n = 18	France<br>n = 125	Germany<br>n = 58	Hungary<br>n = 18	Italy<br>n = 45	Spain<br>n = 31	p-value[a]
**EQ-5D-5L value, mean (SD) using country-specific value sets (this study)**								
**EB type:**								
Dystrophic	0.51 (0.45)	0.75 (0.32)	0.72 (0.23)	0.61 (0.38)	0.57 (0.43)	0.55 (0.39)	0.58 (0.23)	0.167
EB simplex	0.82 (0.13)	0.67 (0.20)	0.72 (0.30)	0.71 (0.35)	0.90 (0.02)	0.70 (0.21)	0.63 (0.27)	0.161
Junctional	0.80 (0.18)	-	0.57 (0.35)	0.74 (0.25)	-	0.61 (0.34)	0.48 (0.22)	0.216
Kindler	0.90 (NA)	-	-	-	0.23 (NA)	0.81 (0.15)	-	0.407
Don't know	0.06 (0.80)	-	0.75 (0.27)	0.00 (NA)	0.56 (0.63)	0.14 (NA)	0.25 (NA)	0.251
**EB severity:**								
Non-severe	0.86 (0.09)	0.78 (0.21)	0.83 (0.22)	0.87 (0.12)	0.80 (0.32)	0.72 (0.26)	0.63 (0.27)	**0.012**
Severe	0.53 (0.47)	0.66 (0.30)	0.62 (0.28)	0.47 (0.40)	0.45 (0.42)	0.51 (0.38)	0.52 (0.21)	0.289
**Total**	0.65 (0.41)	0.71 (0.26)	0.70 (0.28)	0.66 (0.36)	0.59 (0.41)	0.60 (0.35)	0.57 (0.24)	**0.026**
**EQ-5D-5L value, mean (SD) using value sets by Łaszewska et al., 2022[b]**								
**EB type:**								
Dystrophic	0.51 (0.45)	0.75 (0.32)	0.63 (0.27)	0.61 (0.37)	0.65 (0.38)	0.57 (0.35)	0.58 (0.27)	0.476
EB simplex	0.82 (0.13)	0.67 (0.20)	0.63 (0.32)	0.70 (0.34)	0.92 (0.02)	0.69 (0.17)	0.64 (0.29)	0.121
Junctional	0.80 (0.18)	-	0.48 (0.35)	0.73 (0.25)	-	0.64 (0.28)	0.49 (0.27)	0.073
Kindler	0.90 (NA)	-	-	-	0.37 (NA)	0.79 (0.14)	-	0.259
Don't know	0.06 (0.80)	-	0.68 (0.30)	0.00 (NA)	0.64 (0.51)	0.18 (NA)	0.25 (NA)	0.345
**EB severity:**								
Non-severe	0.86 (0.09)	0.78 (0.21)	0.76 (0.25)	0.86 (0.11)	0.84 (0.25)	0.72 (0.24)	0.65 (0.29)	0.058
Severe	0.53 (0.47)	0.66 (0.30)	0.51 (0.30)	0.47 (0.39)	0.55 (0.38)	0.53 (0.32)	0.51 (0.25)	0.548
**Total**	0.65 (0.41)	0.71 (0.26)	0.61 (0.31)	0.65 (0.35)	0.66 (0.36)	0.61 (0.30)	0.57 (0.27)	0.095

[a] Kruskal-Wallis rank sum test, differences between countries.

[b] Value sets by Łaszewska et al., 2022:

- Bulgaria: region = Eastern European
- Germany: region = Central Western
- Austria: region = Central Western
- Spain: region = Southern
- France: region = Central Western
- Hungary: region = Eastern European
- Italy: region = Southern

Łaszewska A, Sajjad A, Busschbach J, Simon J, Hakkaart-van Roijen L. Conceptual Framework for Optimised Proxy Value Set Selection Through Supra-National Value Set Development for the EQ-5D Instruments. PharmacoEconomics. 2022 dic;40(12):1221-34.

Table S4A. Determinants of EQ-5D value

	Univariate model			Multivariate model		
**Variable**	**Beta**	**95% CI**	**p-value**	**Beta**	**95% CI**	**p-value**
**Age ***			0.274			0.292
31-50	0.073	-0.011, 0.156	0.088	0.069	-0.006, 0.143	0.071
51-70	0.076	-0.015, 0.167	0.102	0.055	-0.027, 0.137	0.190
70+	0.048	-0.121, 0.216	0.577	0.090	-0.068, 0.247	0.263
**Sex**			0.556			
Male	-0.022	-0.094, 0.050	0.556	-0.002	-0.060, 0.064	0.948
**Pain**	-0.220	-0.299, -0.141	**<0.001**	-0.114	-0.189, -0.040	**0.003**
**Pruritus**	-0.157	-0.228, -0.086	**<0.001**	-0.057	-0.127, 0.013	0.110
**Skin crusts**	-0.119	-0.192, -0.047	**0.001**	0.009	-0.061, 0.079	0.793
**Atrophic scars**	-0.118	-0.189, -0.047	**0.001**	-0.012	-0.082, 0.057	0.730
**Chronic wounds**	-0.247	-0.312, -0.182	**<0.001**	-0.106	-0.180, -0.032	**0.005**
**Mucosal involvement**	-0.126	-0.195, -0.058	**<0.001**	0.046	-0.028, 0.120	0.221
**Malnutrition**	-0.242	-0.320, -0.164	**<0.001**	0.018	-0.075, 0.111	0.702
**Join deformities**	-0.259	-0.330. -0.187	**<0.001**	-0.097	-0.179, -0.015	**0.021**
**Functional disability**	-0.259	-0.323, -0.194	**<0.001**	-0.099	-0.170, -0.029	**0.006**
**Teeth loss**	-0.206	-0.279, -0.132	**<0.001**	-0.067	-0.146, 0.012	0.094
**Squamous cell carcinoma**	-0.176	-0.277, -0.075	**<0.001**	-0.006	-0.103, 0.092	0.908
**Wheelchair**	-0.410	-0.502, -0.318	**<0.001**	-0.272	-0.367, -0.177	**<0.001**
**Gastrostomy**	-0.242	-0.377, -0.107	**<0.001**	0.026	-0.106, 0.159	0.697
**Esophageal dilatations**	-0.148	-0.232, -0.063	**<0.001**	0.019	-0.067, 0.105	0.667
**Education**			0.126			0.925
Secondary	0.038	-0.092, 0.168	0.564	0.010	-0.101, 0.122	0.853
Tertiary	0.101	-0.026, 0.228	0.120	0.019	-0.089, 0.128	0.727
**Marital status**	-0.073	-0.143, -0.003	**0.041**	0.011	-0.052,0.074	0.728

* Ref. age group 18-30; CI = Confidence Interval; multivariate model R^2 = 0.387; adjusted R^2 = 0.336; F-statistic = 8.854; N = 327

Table S4A. Determinants of EQ VAS

	Univariate model			Multivariate model		
**Variable**	**Beta**	**95% CI**	**p-value**	**Beta**	**95% CI**	**p-value**
**Age ***			0.739			0.943
31-50	1.434	-4.622, 7.491	0.642	0.652	-5.243, 6.547	0.828
51-70	2.311	-4.256, 8.877	0.489	-0.346	-6.826, 6.135	0.916
70+	-4.033	-16.186, 8.120	0.514	-2.973	-15.385, 9.440	0.638
**Sex**			0.059			0.117
Male	-4.920	-10.072, 0.233	0.061	-3.883	-8.745, 0.979	0.117
**Pain**	-11.095	-16.948, -5.243	**<0.001**	-3.790	-9.663, 2.083	0.205
**Pruritus**	-11.639	-16.754, -6.525	**<0.001**	-3.540	-9.041, 1.962	0.206
**Skin crusts**	-11.331	-16.485, -6.176	**<0.001**	-4.801	-10.329, 0.726	0.088
**Atrophic scars**	-7.485	-12.612, -2.357	**0.004**	1.894	-3.639, 7.426	0.501
**Chronic wounds**	-16.567	-21.315, -11.818	**<0.001**	-6.875	-12.727, -1.023	**0.021**
**Mucosal involvement**	-10.192	-15.127, -5.256	**<0.001**	1.127	-4.712, 6.965	0.704
**Malnutrition**	-17.341	-22.952, -11.729	**<0.001**	-3.756	-11.099, 3.586	0.315
**Join deformities**	-14.297	-19.634, -8.959	**<0.001**	-0.950	-7.530, 5.630	0.776
**Functional disability**	-15.617	-20.382, -10.853	**<0.001**	-6.683	-12.263, -1.103	**0.019**
**Teeth loss**	-14.875	-20.176, -9.574	**<0.001**	-5.704	-11.938, 0.530	0.073
**Squamous cell carcinoma**	-10.187	-17.497, -2.877	**0.006**	0.750	-6.925, 8.425	0.848
**Wheelchair**	-13.530	-20.704, -6.356	**<0.001**	-3.026	-10.505, 4.454	0.427
**Gastrostomy**	-18.448	-28.105, -8.792	**<0.001**	-7.920	-18.331, 2.490	0.135
**Esophageal dilatations**	-10.758	-16.798, -4.717	**<0.001**	0.948	-5.830, 7.725	0.783
**Education**			0.069			0.246
Secondary	7.626	-1.832, 17.084	0.114	6.861	-2.013, 15.736	0.129
Tertiary	10.576	1.337, 19.816	**0.025**	7.312	-1.359, 15.982	0.098
**Marital status**	-4.264	-9.305, 0.777	0.097	0.428	-4.560, 5.416	0.866

* Ref. age group 18-30; CI = Confidence Interval; multivariate model R^2 = 0.251; adjusted R^2 = 0.199; F-statistic = 4.817; N = 324.
